# Presence of Macrovolt T Wave Alternans and Short Coupled PVC Simultaneously in a Patient with Long QT Syndrome

**Published:** 2010-04-01

**Authors:** Amir Tavoosi, Abolfath Alizadeh, Mazdak Khalili, Zahra Emkanjoo

**Affiliations:** Department of Pacemaker and Electrophysiology, Rajaie Cardiovascular Medical and Research Center, Iran University of Medical Sciences, Tehran, Iran

**Keywords:** Macrovolt T wave Alternans, long QT syndrome

## Abstract

This report presents a patient with macrovolt T wave alternans, PVC with R on T or a long-short sequence followed by torsades de pointes.

A 35-year-old female was referred to our hospital due to an episode of syncope in the sitting position. This episode was preceded by palpitation and happened two hours prior to our evaluation. There was no history of previous medical problems or drug usage and her physical examination was completely normal. Figure 1 illustrates the 12-lead ECG taken in the emergency room. A few minutes later, she again briefly lost consciousness and recovered spontaneously. Figure 2 demonstrates the rhythm strip recorded by cardiac monitoring during this second syncopal episode.

The ECG in Figure 1 shows normal QRS axis, PR and QRS interval, QTc of 540 msec, and macrovolt T wave alternans best seen in leads I, aVR and V1 [1,2]. There was no precipitant for prolongation of QT interval. Macrovolt T-wave alternans is a harbinger of electrical instability in congenital LQTS, although it could be seen in acquired LQTS [3]. Figure 2 shows a long QT interval and polymorphic ventricular tachycardia (torsades de pointes) that began after a long-short sequence [4]. Two important points regarding this tachyarrhythmia are it's association with macrovolt T wave alternans in the setting of a prolong QT interval and its initiation by a long-short sequence or a PVC during the vulnerable period of the T wave (R on T) [5]. She was managed by implantation of implantable cardioverter-defirillator (ICD) and up titration of propranolol to 40 mg three times a day.

## Figures and Tables

**Figure 1:**
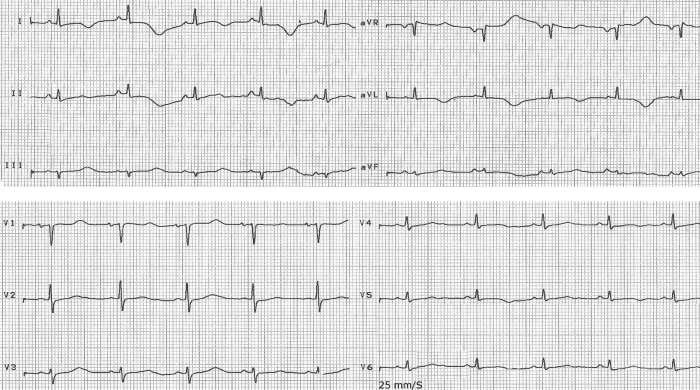
Clearly visible macrovolt T wave alternans and prolonged QT interval in 12-leads ECG immediately before torsades de pointes.

**Figure 2:**
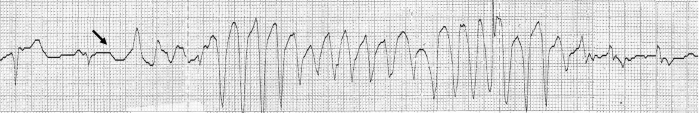
The beginning of torsades de pointes after a short coupled PVC on T wave (arrow).

## References

[R1] Wegener FT (2008). Amiodarone-associated macroscopic T-wave alternans and torsade de pointes unmasking the inherited long QT syndrome. Europace.

[R2] Grabowski M (2004). Drug-induced long-QT syndrome with macroscopic T-wave alternans. Circulation.

[R3] Richard G (1999). Drug-Induced Torsade de Pointes. Circulation.

[R4] Kroll CR (2002). T wave alternans and Torsades de Pointes after the use of intravenous pentamidine. J Cardiovasc Electrophysiol.

[R5] Viskin S (1996). Mode of onset of torsade de pointes in congenital long QT syndrome. Journal of the American College of Cardiology.

